# An Exploratory Study to Develop a Virtual Reality Based Simulation Training Program for Hypovolemic Shock Nursing Care: A Qualitative Study Using Focus Group Interview

**DOI:** 10.3390/healthcare9040417

**Published:** 2021-04-04

**Authors:** Jaehee Jeon, Sihyun Park

**Affiliations:** 1 Department of Nursing, Gangneung-Wonju National University, Gangwon-Do 26403, Korea; anesjjh@naver.com; 2 Department of Nursing, Chung-Ang University, Seoul 06974, Korea

**Keywords:** focus groups, hypovolemia, interview, nursing, shock, simulation, students, virtual reality

## Abstract

Although patients with hypovolemic shock are common in clinical practice, nursing students have little chance of coming across them during their practicum. The main focus of this qualitative study was to explore the elements essential for a virtual reality (VR) based simulation program for hypovolemic shock nursing care. To this end, we conducted focus group interviews with three expert groups of 15 (five from each group) experienced clinical nurses (≥10 years) with experience in hypovolemic shock nursing care. Data were collected in June and July 2020, and after transcribing the interviews, the data analysis involved theme development as part of qualitative content analysis. The exploratory research results were classified into five themes: experience of hypovolemic shock nursing care, determinants of patient prognosis, essential nursing competence, scenario construction, and direction for VR simulation program development. Based on their experience of hypovolemic shock nursing care, the participants suggested concrete development directions for scenarios and VR-based simulation training programs. This article proposes the development of a VR-based simulation program that reflects the exploratory research results of this study in order for nursing students to take an interest in hypovolemic shock nursing care and efficiently improve related skills.

## 1. Introduction

Virtual reality (VR) is a cutting-edge technology that enables a user to experience a realistic life in a computer-generated virtual world [1]. VR programs are interactive simulation systems that allow users to have real-world experiences using computer software and hardware. They can be integrated into a wide variety of settings [2]. Many studies have reported that activities enabled by augmented-VR based on VR programs significantly contribute to improving students’ academic achievements [3,4]. It is successfully applied in various fields of the medical sector, including procedures and surgery, as an integral part of education and training for medical students and doctors [5,6].

With the rapid aging of the population and growing number of severely chronically ill patients, the demand for medical staff with highly specialized knowledge and clinical skills is increasing [7], and continuous on-site presence of healthcare teams is required. Above all, the role of a skilled nurse is of paramount importance in ensuring round-the-clock monitoring of a patient’s symptoms in potentially critical situations and providing prompt care by rapidly recognizing abnormalities and accurately assessing problems [8]. Therefore, in nursing colleges in Korea, nursing students should mandatorily complete at least 1000 h of clinical practicum over four years so that they can perform their clinical duties as skilled nurses after graduation [9]. However, due to recent improvements in patient rights, nursing students cannot directly approach patients [10], and the main part of practicum is observing the nursing care delivered by nurses [9,11]. Therefore, after completing the four-year college course, novice nurses undergo a certain period of reeducation for all nursing tasks, so that they can work independently as nurses [11,12]. Consequently, the workload of senior nurses and the workplace stress of novice nurses increase. This is resulting in an increasingly high nursing turnover rate every year and burdening healthcare providers [12].

Nursing colleges in Korea have been using patient simulation programs in classroom settings for over a decade to address the limitations of observation-oriented clinical practicum [13]. Simulation-based nursing education makes it possible for students to learn how to make an accurate assessment of the patient’s condition, and develop appropriate nursing interventions in complex situations, by creating a realistic clinical environment and applying high-fidelity patient simulators and planned scenarios [14]. In such virtual clinical settings, students can interact with patients with various diseases, understand their respective conditions and symptoms through hands-on experience, and acquire patient interaction skills [15]. However, simulation training has several limitations. For instance, simulated patients are mannequins and even high-fidelity mannequins are far from being like the human body. Moreover, realizable healthcare settings are limited. Finally, nursing activities that can actually be performed on a simulator are limited, and real-time feedback or interaction is virtually impossible [16,17].

Particularly in the field of adult nursing practice, it is difficult to learn how to deal with medical emergency situations by simulating hypovolemic shock from heavy bleeding [10,11]. Under hemorrhagic shock, the patient’s condition rapidly changes. At an earlier stage of blood loss (≤20%), the patient shows subjective symptoms such as anxiety and restlessness. Physical symptoms, such as tachycardia, tachypnea, oliguria, and orthostatic hypotension, appear in the medium stage of blood loss (20–40%), possibly accompanied by symptoms suggestive of internal hemorrhage, such as hematemesis, bloody stool, and abdominal distention. When the blood loss exceeds 40%, various systemic reactions, such as hypotension, severe tachycardia, and deteriorating mental state, begin to manifest [18]. Considering these different stages of hemorrhagic shock and their respective symptoms, a nurse should immediately recognize the patient’s condition and plan and implement appropriate nursing interventions [18]. Medical emergency situations such as hypovolemic shock are common clinical cases, and the patient’s safety can be determined by the speed and efficiency of the nursing care provided. Therefore, it is necessary to prepare nursing students for medical emergency situations by providing them with an efficient simulation-based nursing program or course capable of simulating emergency situations, including hypovolemic shock. However, existing simulation training has limited application in simulating real situations.

VR is used extensively in the medical sector for education and patient treatment. VR-based simulation education has the advantage that nursing students can learn through autonomous and repetitive intervention experiences [15]. There have been attempts to develop VR-based simulation training systems in the field of nursing [19,20], but this is still in its infancy. Therefore, this study focuses on exploring the elements that must be included in a VR-based simulation training program for hypovolemic shock nursing care to be developed for nursing students. To this end, we conducted a focus group interview with experts having at least 10 years of clinical experience, including nursing care for patients with hypovolemic shock.

## 2. Materials and Methods

### 2.1. Study Design

This study utilized a qualitative design and data were collected with the help of focus group interview.

### 2.2. Sample

The inclusion criteria for the participants in this study were (i) nursing experts with experience in hypovolemic shock nursing care, and (ii) a clinical career of at least 10 years [21] in internal medicine wards, intensive care units, or emergency rooms.

Snowball sampling was employed to recruit participants, beginning with the initial participants recommended by each of the three different general hospitals. The snowball participant recruitment continued until 15 nursing experts, five from each group, were gathered. This process involved switching between data collection and analysis. No further recruitment was necessary because the interview data collected from the third focus group interview were judged to have reached theoretical saturation, covering all topics necessary for the intended purposes.

### 2.3. Procedure

To obtain Institutional Review Board (IRB) approval, we asked the heads of nursing departments of the three general hospitals, where recruitment took place, for consent and cooperation. The data collection began through a focus group interview with the participants who gave voluntary informed consent. Data were collected in accordance with Breen’s guidelines [22] for focus group interviews. The data collection guide is presented in Table 1.

#### 2.3.1. Interview Questions

Prior to the interview session, a set of draft focus group questions was developed by the research team based on literature review and discussion. According to Breen’s guidelines [23], they were organized by progressiveness. The key interview questions used in the focus group interviews are presented in Table 2.

#### 2.3.2. Interview Procedure

A pre-test was conducted before the interview. In the pre-test, we checked the interview questions, the interview process, and the environment in which data were collected. Regarding the interview questions, the appropriateness of the interview questions and the difficulty in answering the questions were confirmed with the two research assistants working as a nurses. The research assistant suggested that the opening question should determine whether the participant had experience with VR and add it accordingly. They said it was not particularly difficult to answer other questions. After visiting various places for an appropriate environment, an independent, quiet, and unobstructed study room was reserved in advance. For the interview process, the data collection guide was checked, and the necessary data on the day of the interview were additionally prepared considering the variability of the target group. In addition, the researchers practiced the questions before the actual interview.

Data were collected in June and July of 2020.

A quiet study room was rented as the venue for the focus group interviews. Refreshments were prepared for the study participants. After the arrival of all participants, explanation sheets were distributed. After detailed explanations about the purpose of the study, interview procedure, and data management and disposal, written consent was obtained from each participant.

The moderator and assistant moderator were introduced prior to the main interview session. The participants were assured that all information derived from the interview session would be used exclusively for study purposes, and the recording began after all participants agreed to the session being recorded.

In the course of the session, interview questions were presented, and participants were invited to a free discussion. The session lasted about two hours until no new content emerged regarding the topics presented. Each participant was given sufficient time to provide an answer from their own experiences and perspectives. During the session, participants were encouraged to feel free to express their opinions through non-verbal communication skills such as nods and smiles.

In the wrap-up phase, all participants were given an additional opportunity to add anything they had in mind. The moderator thanked the participants and offered souvenirs as a token of appreciation. 

### 2.4. Ethical Consideration

This study obtained IRB approval (No. GWNUIRB-2020-3) from the university to which the principal researcher was affiliated. Prior to the focus group interview session, the participants were informed of their right to withdraw their participation at any time, even during the session, and of the confidentiality of the interview content along with their anonymity. In the transcription process, all personal information of the participants was anonymized and any identifying content was redacted. All research data will be stored under lock for three years and disposed of properly.

### 2.5. Data Analysis

Data analysis involved a qualitative content analysis [23]. Before proceeding to the stage of developing a theme in this analysis, the recorded interviews were transcribed by a research assistant and confirmed by the research team. 

The first step of the analysis was the initialization phase. The research team repeatedly read the transcript, underlined meaningful answers for major questions, and attempted to fully understand the meaning and contents. The second step was the construction phase wherein the extracted meaningful statements and main contents were classified. Similar and different contents were classified, compared, and then coded. Clusters of similar codes were labeled, translated, defined, and described. The third step was the rectification phase. In this step, the theme was on the verge of full development. It involved checking, confirming, and ensuring the relative certainty of the developed themes. The last step of theme development was the finalization phase. A narration developed by the researchers as a written commentary described and connected the various themes and answered the study question.

### 2.6. Validating the Concept of Experience and Validity Test

The theoretical model for verifying the concept of experience on the subject is based on the postpositivist paradigm. The paradigm of postpositivism uses credibility, transferability, dependability, and confirmability for quality management of qualitative research [24]. In this study, the researcher lens, the research participant lens, and the external evaluator lens [25], which are the viewpoints used by the researcher to establish validity, were used to secure them. First, the researcher lens is the main lens that determines the reliability of qualitative research, and the qualitative researcher who hired the researcher lens continuously refers to the original data to see if the subject, classification, explanation, and interpretations derived from their analyses are understandable, and should be reviewed [26]. In this way, the interaction between the researcher, the subject, and the process of making it understandable is called the validity as a reflective explanation [27]. In this study, triangulation was used as the researcher’s lens method. In other words, at the time of the interview, one researcher led the interview, and the other researcher observed and recorded the behavior of the researcher and the research participant, and the research environment for the entire interview process. Second, the research participant lens suggested the importance of examining how accurate the reality recognized by the study participants was reflected in the final description [26]. In this study, member checking was performed. After transcription of the interviewed data, participants were asked to check whether the contents of the manuscript were answered correctly or if there was any comment. Third, the lens of the external evaluator commissioned an audit trail of the entire contents of the research interview, such as the results and the technology, by an external expert who was not directly related to the research. External experts were two nursing professors with more than 10 years of nurse experience in qualitative research experience. 

The qualitative validity of the study was verified in accordance with the four criteria for qualitative research created by Lincoln and Guba [24]: credibility, fittingness, auditability, and confirmability. Credibility was achieved by listening to the recorded content repeatedly, checking it against the transcribed content to ensure its accuracy, and having the content of any inaccurate or ambiguous part confirmed or rectified by the corresponding participant. Fittingness was achieved by selecting participants suitable for the research objectives and continuing the focus group interview until the theoretical saturation of the research objectives. Auditability was achieved by constituting the researcher team with qualified professionals capable of comprehensively reviewing and verifying the transcribed and analyzed contents, and by having an external qualitative research expert perform an overall review. Confirmability was achieved by recording the entire process of data collection and storing the recorded and transcribed data for inspection at any time.

## 3. Results

### 3.1. General Characteristics of the Participants

All participants were female nurses (ages 35–49 years). The average number of years of clinical career was 22 (Table 3).

### 3.2. Content Analysis Results

The research team comprehended the meaning of the transcribed data of the interview as a whole, and set three criteria for the extraction of analysis units: “experience of hypovolemic shock nursing care,” “simulation training scenarios for hypovolemic shock nursing care,” and “development of VR-based simulation training program for hypovolemic shock nursing care”.

According to the criteria, we extracted 112 units of analysis, which were then open-coded into three larger codes (i.e., experience of nursing care, scenario, and simulation training program) and six smaller codes (i.e., experience of hypovolemic shock nursing care, difficulties with nursing care, nursing competence, direction of education when teaching through simulation training, scenarios, and development direction of VR-based simulation training program). 

The exploratory results for the development of a VR-based simulation training program for hypovolemic shock nursing care were verified through the process of categorizing the coded analysis units and analyzing the system within the category and abstraction. They were categorized into five themes (experience of hypovolemic shock nursing care, determinants of patient’s prognosis, essential nursing competence, scenario construction, and direction of VR-based simulation training program; Table 4).

#### 3.2.1. Theme 1: Experience of Hypovolemic Shock Nursing Care

Participants did not know what to do in the face of the sudden patient emergency situation and felt the limit of their role as a nurse in the process of initial assessment of the patient’s condition and coming up with proper interventions. On the other hand, they felt a sense of pride when the patient’s condition improved thanks to their prompt discovery of the situation and efficient teamwork in coping with the situation. This theme comprises the following categories:

(1) Being at a loss for what to do

This category comprises the following six extracted codes. When early-stage symptoms of hypovolemic shock manifested, participants were not aware that they were associated with the emergency situation of hypovolemic shock. They faced difficulties such as in providing treatment and keeping nursing records in an emergency situation. They therefore showed disoriented actions in a state of total confusion, feeling the limits of their coping ability. Above all, they realized in this process that the patient’s condition could be much more serious than indicated by their initial symptoms.

‘A novice nurse was caring for a postoperative patient. She was emptying the Hemovac drain every hour, 200–300 mL, and recording it without giving a thought to the bleeding. After a few hours, the patient suddenly went into shock.’

(Participant A)

‘During the night shift, there were not enough nursing staff. The patient suddenly developed hematemesis in the ward, and the condition worsened. Two nurses panicked and made an emergency call ... they were barely following the doctor’s orders, without even thinking of recording the situation.’

(Participant C)

(2) Limited nursing care

This category comprises the following two extracted codes. Participants were busy following doctors’ orders in an emergency situation wherein a patient went into hypovolemic shock. All situations were determined by the test results, equipment, and doctors, with the nurses’ role being limited.

‘The equipment quality has improved significantly in recent years. If a patient is suspected of having a certain condition, sonography is performed immediately to determine the cause and proceed with immediate intervention treatment. The circumstances for the nurse to intervene are very limited.’

(Participant E)

‘Patients who are presented with hypovolemic shock don’t have consciousness in the first place, so there is not much that can be done by the nursing team. Especially in the emergency room... the doctor immediately checks the patient and gives instructions, and the nurses are busy following the orders.’

(Participant H)

(3) Sense of pride

This category comprises the following five extracted codes. Participants accurately assessed patients with hypovolemic shock, reported it to the doctor appropriately, and promptly coped with the situation. In an emergency, their superhuman ability was instantly discovered, and as a result, they were thrilled during patient recovery.

‘I was caring for a patient undergoing liver transplantation. When monitoring the vital signs, I found the pulse rate to be elevated. Alarmed by my strange feeling, I carefully checked the urine output and the color and amount of fluid in the hemovac drain. Abdominal distension was also observed, and bleeding at the surgical site was suspected. Accordingly, I immediately notified the doctor ... Immediately after the examination, a full drop of transfusion and saline was dispensed, and the patient underwent emergency surgery... The patient’s condition improved.’

(Participant N)

‘Teamwork is the key point in such a situation. If you coordinate well with your team nurse, you can solve it well.’

(Participant B)

#### 3.2.2. Theme 2: Determinants of Patient’s Prognosis

In the process of early identification and treatment, participants realized the importance of the role of an excellent nurse and environmental requirements such as preparing a checklist for possible emergency situations and boosting teamwork with colleagues. This theme comprises the following categories:

(1) Early assessment and prompt treatment

This category comprises the following two extracted codes. Participants emphasized that the golden time for a positive prognosis of patients with hypovolemic shock is the earliest possible identification of the symptoms, rapid identification of the cause and corresponding treatment. They discussed that they were able to understand the types of diseases that can cause hypovolemic shock and discern the degree of shock progression by identifying various early symptoms of each underlying disease.

‘It is usually a nurse that detects symptoms because nurses are always near patients. Although the vital signs are objective, they may not change significantly during the initial shock phase. Therefore, it is important to check for symptoms such as a patient’s complexion change, anxiety, and nausea.’

(Participant F)

‘It seems that the golden time to save the patient is the early detection of hypovolemic shock and rapid treatment matching the cause.’

(Participant H)

(2) Excellent individual nursing skills

This category comprises the following five extracted codes. Participants experienced that a patient recovered faster when cared for by an excellent nurse capable of discovering the initial symptoms and responding quickly and efficiently by easily controlling the progression using a rich knowledge base and experience in such clinical situations. Nurses should have a basic understanding of drugs, fluids, and electrolytes that are commonly used in emergency situations.

‘Symptoms or causes of hypovolemic shock may differ depending on the underlying disease. The more experienced a nurse is, the greater the difference is in identifying such disease-specific symptoms compared with novices. Such nurses respond to emergency situations with dazzling accuracy and speed.’

(Participant K)

‘It is important to rapidly administer the correct fluid prescribed by the doctor from a large variety of fluids. Ideally, a nurse should be able to raise a well-informed question when a prescription is inadequate for the patient’s condition.’

(Participant I)

(3) Environment ready for coping with emergency situation

This category comprises the following three extracted codes. Participants recalled that the use of a checklist with all possible emergency situations that may be caused by the sudden occurrence of hypovolemic shock was highly efficient. In this respect, they also recalled the positive outcomes achieved by solving the situation through teamwork with appropriate task allocation among the team of nurses.

‘The nursing department prepared manuals and checklists to help nurses respond to emergency situations, such as shock, and trained them routinely according to those manuals and checklists. In this way, nurses are better prepared when such emergency situations occur.’

(Participant G)

‘When a sudden hypovolemic shock occurs, nurses run to the patient. Then, instant task assignment occurs. One nurse is in charge of charting, another oxygen supply, and a third medication check ... Good task assignment and implementation is important for the success of nursing care.’

(Participant D)

#### 3.2.3. Theme 3: Essential Nursing Competence

Participants agreed that the essential nursing skills necessary for hypovolemic shock nursing care are the ability to identify the patient’s condition as early as possible and cope with the situation from various aspects. This theme comprises the following categories:

(1) Judgment and assessment of the situation

This category comprises the following four extracted codes. Participants considered it necessary for nurses to have the ability to respond sensitively to changes in vital signs when they detect a patient in hypovolemic shock, and to confirm the patient’s condition objectively through necessary tests and results after that. To objectively check the patient’s condition, it is necessary to be able to interpret blood test results, start electrocardiogram (EKG) monitoring quickly, and interpret the results.

‘The first thing you learn at a nursing college is measuring the vital signs. Ironically, however, maybe because it is done too much and common, they seem to forget the importance of vital signs.’

(Participant A)

‘When it comes to hypovolemic shock, we instantly think of vital signs. In reality, however, patients have a variety of typical symptoms, starting from coldness. Such a first symptom alarms a skilled nurse, who then concurrently assesses other typical symptoms such as sweating and restlessness, which should be evaluated simultaneously.’

(Participant H)

(2) Nursing care for shock patients

This category comprises the following three extracted codes. Participants noted that once the hypovolemic shock state is confirmed, nurses are required to have the knowledge and skills to rapidly administer appropriate treatment in collaboration with the team members. Knowledge and skills include an understanding of emergency medications and the ability to competently use the necessary medical devices.

‘Hospitals use a wide range of medical equipment. First, novice nurses have difficulty handling them because they are unfamiliar with them. Considering that students are skilled with smartphones and computers, they should be trained to handle medical equipment such as EKG monitors and infusion pumps in clinical practice.’

(Participant F)

‘Nurses need to have good knowledge of emergency medication because they have to move quickly... Without prior knowledge, they are prone to panic in emergency situations.’

(Participant I)

#### 3.2.4. Theme 4: Scenario Construction

The participants emphasized the importance of constructing hypovolemic shock scenarios with postoperative patients and concluded with the final scenario of positive patient outcomes. This theme comprises the following categories:

(1) Topics of hypovolemic shock scenarios 

This category comprises the following three extracted codes. Participants admitted the necessity of nursing students to experience shock cases caused by various underlying diseases but recommended first and foremost the construction of scenarios for emergency room (ER)-related postoperative patients.

‘I think an ER setting will be of more help to the students because it may be more interesting and challenging to detect a hypovolemic situation through physical examination in an undiagnosed state.’

(Participant J)

‘Hypovolemic situations may be caused by a variety of conditions, such as trauma, variceal bleeding, and postoperative situations. I think it would be of great help to acquire basic knowledge of each of these situations, and I hope that nursing students receive related training at college.’

(Participant A)

(2) Final positive patient outcome

This category comprises two extracted codes. Participants recommended that students should experience a positive patient outcome in emergency situations in the simulation training of hypovolemic shock. It is because they want the student to not get disheartened or not be nervous while going into the situation.

‘Ending should be positive, similar to cardiac massage or CPR [cardiopulmonary resuscitation]. Otherwise, sending the patient to the operating room or seeing the symptoms stabilizing...’

(Participant F)

‘First, I think it too dramatic to have the patient expire as the scenario ending.’

(Participant I)

#### 3.2.5. Theme 5: Direction of VR-Based Simulation Training Program

Participants suggested that when developing a VR-based simulation training program for hypovolemic shock nursing care, scenario-based algorithms and necessary checklists should be created. Optional features should also be provided for the students to choose from, according to various training situations. Participants also suggested that directions should be given for concrete assessment of the patient’s condition and for judgment about various interventions. Above all, a program should be developed in a way that generates students’ interest so that they can engage in training with more enthusiasm. This theme comprises the following categories:

(1) Algorithms and checklist

This category comprises the following three extracted codes. Participants recommended the application of overall algorithms to the program so that students could experience different outcomes or paths depending on their decision-making regarding the assessment of hypovolemic situations and corresponding interventions.

‘When VR is turned on, various optional features should appear. Options might also be presented for different diseases, and disease-specific options for questions may also be provided. Symptom assessments will also have to be provided as options.’

(Participant J)

(2) Option setting tailored to the situation

This category comprises the following five extracted codes. Participants recommended that options be provided for initial symptoms shown by patients with hypovolemic shock so that students can identify various initial symptoms, verify their causes, and proceed with interventions accordingly. They also proposed to include an implementation function in which stepwise treatment according to the doctor’s orders is provided.

‘The degree of difficulty should be divided into basic and advanced courses, whereby the basic course requires basic interventions once the shock is confirmed, such as raising the patient’s legs, securing the intravenous line, and performing fluid changes, as well as related medication and execution of CPR.’

(Participant A)

‘For example, hematochezia and hematemesis are visible, but melena may develop unnoticed by the patient. In this context, more detailed questions such as “Did you see blood on the toilet paper after wiping?” seem to help students really.’

(Participant F)

‘If the doctor gives orders, it would also be helpful to provide options for implementation.’

(Participant I)

(3) Provision of the direction of assessment and intervention

This category comprises the following seven extracted codes. Participants provided ideas for the assessment of the condition of a patient with hypovolemic shock and intervention options in various directions based on their clinical experience.

‘I tell the students that these three points must be considered when they see a patient in shock: change in mental alertness, urine output, complexion change...but the urine output is difficult to check right away. Then you should see to it that a Foley catheter be placed.’

(Participant G)

‘It would be fun if a specific situation were given; for example, postpartum bleeding in obstetrics and gynecology... For it is a critical emergency situation, the amount of blood loss must be estimated quickly, and a proper transfusion regimen should be applied as soon as possible... I think repeated training in such concrete situations would help nursing students when they work later as nurses.’

(Participant L)

(4) Interest-inducing AI-based learning

This category comprises the following two extracted codes. The VR-based simulation training program should be brief for optimum levels of attention and interest. The participants also highlighted the importance of keeping records and writing reports about the process and outcomes of the VR-created clinical situations.

‘I had different VR experiences earlier, but when the time got longer, I could not concentrate and had symptoms such as nausea and dizziness. Therefore, it would be nice if the time did not exceed 15 min.’

(Participant D)

‘Nowadays, students lose interest when they are bored. They are the IT generation. Therefore, I believe that the efficient application of VR would induce them to learn with more interest. It is all the more necessary as they cannot participate in clinical practicum because of COVID-19.’

(Participant H)

## 4. Discussion

This exploratory study aimed at exploring the elements necessary for developing a VR-based simulation training program for hypovolemic shock nursing care. The results were derived from a focus group interview with nursing experts who frequently experienced hypovolemic shock cases in actual clinical settings. During the interview session, the participants’ clinical experiences with patients with hypovolemic shock were verified. Based on their experiences, they proposed the determinants of patient prognosis and the directions for constructing educational scenarios for nursing students. They also provided various ideas for the development of a VR-based simulation training program, including essential nursing skills that the program should help develop to care for patients with hypovolemic shock.

Some of the participants’ experience of caring for patients with hypovolemic shock began with being at a loss for what to do in the face of a sudden hypovolemic shock situation and realizing the limited role of a nurse, but some participants also felt a sense of price when the patient’s condition improved due to their correct assessment and prompt reporting. Although there is no research dedicated to the experience of hypovolemic shock nursing care, a study described a case of nurses’ failure to cope properly with emergency situations requiring CPR. This was because the nurses were unfamiliar with emergency management and the suddenness of the critical situation led them to panic [28]. On the other hand, a study reported successful cases where novice nurses rapidly assessed the patient’s condition in a clinical emergency and administered appropriate interventions, resulting in positive patient outcomes. This further improved novice nurses’ clinical skills and confidence in nursing care [29]. Therefore, it is of crucial importance to provide guidance and proper training to nursing students so that they can successfully deal with emergency situations, such as hypovolemic shock, in actual clinical settings.

Early assessment and prompt treatment, excellent individual nursing skills, and an environment ready for coping with emergency situations were identified as factors associated with the prognosis of patients with hypovolemic shock as experienced and described by the participants. Many studies have also verified that active and appropriate interventions based on rapid and accurate assessment of initial symptoms contributed to the positive prognosis of patients in shock [30] and reduced morbidity and mortality [31]. The role of nurses was particularly emphasized [32], as was the importance of hospital systems, education, and training [29]. 

Proper judgment of the situation, rapid assessment of the patient’s condition, and efficient response are the nursing skills necessary for hypovolemic shock nursing care as experienced by the participants. The incidence of hypovolemic shock is common in clinical practice, but it is a situation which nursing students have little chance of experiencing directly [10]. In fact, since novice nurses do not have sufficient clinical experience in various emergency situations, they often fail to respond appropriately to the sudden occurrence of emergency situations and suffer from increased mental burden and stress [33]. Therefore, when constructing a simulation training program for hypovolemic shock nursing care to educate nursing students, it is necessary to incorporate the essential elements suggested by the results of this study.

Based on their own experience of hypovolemic shock nursing care, the participants identified the determinants of patient prognosis and essential nursing skills and suggested ideas for constructing simulation training scenarios based on the factors identified. As topics for the simulation training scenarios, the participants suggested contradictory scenarios such as making students experience various diseases found in ER settings or providing less complex cases of postoperative patients. However, they agreed on providing positive patient outcomes when concluding simulation training. In related literature, well-organized emergency nursing simulation education was able to improve the academic achievement, self-efficacy, and learning attitude of nursing students [13], with strategies to enhance academic achievement and self-efficacy, in particular, occurring in the process of self-directed solving of the presented problems [34]. In light of these findings of previous studies, it is necessary to construct scenarios with topics that allow easy solutions to changing situations in a simple setting rather than a complex setting. Scenarios should be simple enough that they can be solved by nursing students at their level of knowledge and skills.

Participants suggested specific directions for developing the envisaged VR-based simulation training program for hypovolemic shock nursing care. First, they proposed the development of algorithms and checklists for each of the detailed stages, such as the overall situation, assessment, and intervention, and the configuration of various concrete options for the implementation of the program. Next, they suggested the range and characteristics of instructions to guide the students in improving the knowledge necessary for assessing the patient’s condition and administering appropriate interventions. In addition, it was suggested that the program should be kept brief (preferably less than 15 min) in order to keep the students immersed in the scenarios and avoid any side effects of using VR for too long. In previous studies as well, in order to develop VR-based nursing education simulation, construction of a flow chart and concretely designed block diagrams were recommended for all processes to better achieve the program requirements or goals [35]. Furthermore, it was seen that despite VR-based nursing simulation training’s various advantages, such as enhanced self-efficacy and communication ability [36], VR-induced physical side effects, such as nausea and dizziness, have also been reported, [37]. Therefore, various aspects must be considered when developing VR-based nursing student training programs.

Simulation training using VR will gradually expand in clinical practice education for nursing students, provided impetus by limitations of the current training methods and environments as well as changing times. While the present study aimed to find new developments in this direction using in-depth focus group interview, it has a limitation in that its objective aspect can be inferior to that of quantitative research. However, it has the benefit of being an in-depth qualitative study. The development of a VR-based simulation training program requires active scenario-based intervention by clinical experts to ensure its practicality and accessibility, considerations of the needs of its end users, and the overall collaboration process in the IT field to ensure a smooth technical implementation [38]. This study is significant in that it presents realistic and concrete ideas for a group of experts. Therefore, when adequately integrated and applied, the results derived in this study are expected to contribute to developing a VR-based simulation training program for hypovolemic shock nursing care. 

## 5. Conclusions

This exploratory study aimed to provide basic data for developing a VR-based simulation training program for hypovolemic shock nursing care based on a focus group interview with nursing experts with experience of hypovolemic shock nursing care. The analysis results of the in-depth focus group interview were classified into five categories (experience of hypovolemic shock nursing care, determinants of patient’s prognosis, essential nursing skills, scenario construction, and direction of VR-based simulation training program), 14 thematic clusters, and 49 themes.

When developing a VR-based simulation training program for hypovolemic shock nursing care for nursing students, it is necessary to provide concrete stepwise algorithms and optional features in the overall composition and detailed plan. A simple case, such as a postoperative patient, is recommended as the cause of hypovolemic shock. Particular care should be taken at the design stage to provide nursing students with opportunities to acquire the knowledge and skills necessary for identifying initial symptoms and executing various interventions. It is also necessary to set an appropriate time frame and create an environment that appeals to students so that they are ready to learn with interest.

## Figures and Tables

**Table 1:** Data collection guide.

**1.** **Preparation for interview**	**2.** **Preparing the interview location**
Data: Location: Participants: Arrive 15 min before the interview time Preparation of materials - Research Manual and Participation Agreement - Demographic characteristics document - Interview guidelines - Recorder - Check the battery - Clock - Pen - Field Records - Extra paper - Participant present	Table Chair Tea or coffee, biscuits, water Tissue
**3.** **At the start of the interview**	**4.** **At the end of the interview**
- Explain the purpose of the study - Description of the participant selection process - Interview precautions - Obtain written consent from research participation - Turn off the mobile phone - Noise reduction	If necessary, check whether additional interviews are possible

**Table 2:** Questions for the focus group interview.

Categories	Questions
Opening questions	- Have you ever cared for a patient with hypovolemic shock? - Do you know of simulation training for nursing students? - Have you ever experienced VR?
Introductory questions	- What was your experience with patients with hypovolemic shock?
Transition questions	- What are the nursing skills required for hypovolemic shock nursing care? - What are the most important nursing skills for hypovolemic shock nursing care? - What were the difficulties you encountered during hypovolemic shock nursing care?
Key questions	- What should nursing students learn to be able to provide good nursing care for patients with hypovolemic shock? - Based on your experience, what scenarios should be included in a VR-based simulation training to train nursing students for hypovolemic shock nursing care? - What are the necessary elements when developing a VR simulation training program for hypovolemic shock nursing care?
Ending question	- Do you have anything else to say?

**Table 3:** Demographic characteristics of participants (N = 15).

ID.	Age	Position	Total Clinical Experience (Years)	Experience of Medical-Surgical Ward (Years)	Experience of Incentive Care Unit (Years)	Experience of Emergency Room (Years)
A	46	Head nurse	23	2	16	
B	42	Floor nurse	19	13	6	
C	56	Head nurse	20		16	
D	49	Head nurse	28		22	4
E	44	Floor nurse	21			13
F	51	Head nurse	21		11	5
G	35	Staff nurse	13		13	
H	41	Floor nurse	19	2	6	
I	47	Floor nurse	23		13	
J	49	Floor nurse	27	5	22	
K	49	Head nurse	27	5	15	
L	47	Floor nurse	25		23	2
M	48	Floor nurse	26		9	
N	46	Head nurse	25		20	
O	37	Staff nurse	13		13	

**Table 4:** Results of the content analysis to develop a virtual reality (VR) based simulation program for hypovolemic shock nursing care.

Extracted Code	Categories	Themes
Unaware of the emergency situation Suddenness of the situation leading to confusion and disoriented actions Feeling of the limits of coping ability Unaware of the progress of emergency situation Difficulty keeping nursing records in emergency situation Realizing that there are more than visible phenomena	Being at a loss for what to do	Experience of hypovolemic shock nursing care
Limited scope of manageable nursing Greater role of doctors and equipment	Limited nursing care	
Quick discovery of the situation Realizing the importance of teamwork with colleagues Importance of accurate assessment and proper reporting Spontaneous display of superhuman ability Feeling exulted at the recovered patient’s condition	Sense of pride	
Golden time of early assessment and adequate treatment Understand the differences in initial assessment approaches for individual diseases	Early assessment and prompt treatment	Determinants of patient’s prognosis
Patient’s prognosis dependent upon nursing skills Basic knowledge about drugs, fluids and electrolytes	Excellent individual nursing skills	
Usefulness of emergency checklist Importance of teamwork while dealing with crisis Prompt and appropriate division of work among colleagues	Environment ready for coping with emergency situation	
High importance of measuring vital signs and results Quick and objective assessment of early symptoms Necessity of blood test and link between result and treatment Monitoring and interpreting of the electrocardiogram (EKG)	Judgment and assessment of the situation	Essential nursing competence
Proficient use of medical devices Knowledge of emergency medication Prompt work allocation and cooperation with colleagues	Nursing care for shock patients	
Experiencing various situations with hypovolemic shock Experiencing hypovolemic shock nursing care in an emergency room Application of post-operation case	Topics of hypovolemic shock scenarios	Scenario construction
End with a positive patient status Resolved ending	Final positive patient outcome	
A step-by-step checklist for assessment and intervention Systematization of algorithms for situations and treatments Step-by-step application of real-time treatment interventions	Algorithms and checklist	Direction of VR-based simulation training program
Provision of multiple choices for selecting early symptoms Setting of disease-specific options for initial symptoms Providing cause-based treatment options Providing scenarios and options of different difficulty levels Providing step-by-step treatment according to order	Option setting tailored to the situation	
Objective assessment tips for characteristic early symptoms Securing emergency injection route Situation requiring EKG monitoring Repetitive practice of interventions on specific situations Practice on how to use various medical devices Understanding of drugs used in emergency situations Training on the use of emergency drugs	Provision of the direction of assessment and intervention	
Keep the VR-created clinical situation brief Develop prompt and detailed reporting skill	Interest-inducing artificial intelligence-based learning	
